# A novel role for farnesoid X receptor in the bile acid‐mediated intestinal glucose homeostasis

**DOI:** 10.1111/jcmm.15881

**Published:** 2020-10-08

**Authors:** Long Zhao, Zefeng Xuan, Wenfeng Song, Shiyu Zhang, Zequn Li, Guangyuan Song, Xingxin Zhu, Haiyang Xie, Shusen Zheng, Penghong Song

**Affiliations:** ^1^ Division of Hepatobiliary and Pancreatic Surgery Department of Surgery First Affiliated Hospital School of Medicine Zhejiang University Hangzhou China; ^2^ NHCPRC Key Laboratory of Combined Multi‐organ Transplantation Hangzhou China; ^3^ Key Laboratory of the Diagnosis and Treatment of Organ Transplantation CAMS Hangzhou China; ^4^ Key Laboratory of Organ Transplantation, Zhejiang Province Hangzhou China; ^5^ Collaborative Innovation Center for Diagnosis Treatment of Infectious Diseases Hangzhou China

**Keywords:** bile acid, FXR, glucose homeostasis, GLUT2

## Abstract

The farnesoid X receptor (FXR), as a bile acid (BA) sensor, plays an important role in the regulation of lipid metabolism. However, the effects and underlying molecular mechanisms of FXR on intestinal glucose homeostasis remain elusive. Herein, we demonstrated that FXR and glucose transporter 2 (GLUT2) are essential for BA‐mediated glucose homeostasis in the intestine. BA‐activated FXR enhanced glucose uptake in intestinal epithelial cells by increasing the expression of GLUT2, which depended on ERK1/2 phosphorylation via S1PR2. However, it also reduced the cell energy generation via inhibition of oxidative phosphorylation, which is crucial for intestinal glucose transport. Moreover, BA‐activated FXR signalling potently inhibited specific glucose flux through the intestinal epithelium to the circulation, which reduced the increase in blood glucose levels in mice following oral glucose administration. This trend was supported by the changed ratio of GLUT2 to SGLT1 in the brush border membrane (BBM), including especially decreased GLUT2 abundance in the BBM. Furthermore, impaired intestinal FXR signalling was observed in the patients with intestinal bile acid deficiency (IBAD). These findings uncover a novel function by which FXR sustains the intestinal glucose homeostasis and provide a rationale for FXR agonists in the treatment of IBAD‐related hyperglycaemia.

## 1 INTRODUCTION

Bile acids (BAs) are amphipathic steroid molecules that facilitate dietary lipid absorption and cholesterol catabolism.^1^, ^2^ It not only activate nuclear receptor farnesoid X receptor (FXR, NR1H4) and the G protein‐coupled receptor TGR5, but also participate in regulating multiple protein kinase pathways, such as the protein kinase A/C and mitogen‐activated protein kinases (MAPKs) pathways.^3^, ^4^ Alterations in the amount or composition of BAs result in a range of metabolic disease such as diabetes, obesity and non‐alcoholic fatty liver disease.^1^, ^5^, ^6^, ^7^ However, the role of BAs in the intestinal glucose homeostasis and its underlying regulatory mechanisms are unclear and remain to be elucidated.

FXR is the primary BA biosensor that mainly expressed in the liver and intestine. It plays a critical role in the regulation of lipid and BA metabolism.^2^, ^8^, ^9^ Recently, few studies suggest that FXR controls carbohydrate metabolism through the regulation of glucose tolerance and insulin sensitivity.^10^, ^11^, ^12^ FXR knockout mice exhibit impaired glucose tolerance and hyperglycaemia when fed on a normal chow diet,^10^, ^12^ whereas activation of FXR is thought to be beneficial for the treatment of patients with diabetes and abnormal glucose metabolism.^7^, ^13^ Despite the discovery of a pivotal role of FXR in regulating glucose metabolism, it remains unknown whether FXR affects glucose metabolism through modulation of intestinal glucose homeostasis.

In the small intestine, the transepithelial transport of glucose is mainly mediated by the apical sodium‐glucose transporter 1 (SGLT1) and basolateral glucose transporter 2 (GLUT2) in the intestinal epithelial cells.^14^, ^15^ In response to high luminal glucose, GLUT2 is recruited to the apical membrane, allowing the mass transport of glucose into the blood,^16^, ^17^ which is associated with the SGLT1‐dependent activation of PKC βII and MAPK signalling pathways.^17^, ^18^ SGLT1 or GLUT2 knockout mice exhibited impaired intestinal glucose absorption and decreased blood glucose level.^19^, ^20^ Although multiple factors, including metabolic enzymes, hormones, intestinal microbiota and drugs, regulate glucose metabolism by altering the expression or distribution of the above glucose transporters,^21^, ^22^, ^23^ it remains poorly understood how the activation of FXR regulates glucose transporters in the intestine.

In this study, we demonstrate that the effect of FXR on the BA‐mediated glucose homeostasis in intestinal epithelial cells is a dynamic equilibrium process. BA‐activated FXR not only contributes to the increase of GLUT2 expression for controlling glucose uptake through the FXR‐S1PR2‐ERK1/2 signalling cascade, but also reduces the cell energy generation via inhibition of oxidative phosphorylation (OXPHOS), which is crucial for intestinal glucose transport. Meanwhile, BA‐mediated FXR inhibits transepithelial transport of glucose by altering the ratio of GLUT2 to SGLT1 abundance in the intestinal brush border membrane (BBM). Notably, the impaired intestinal FXR signalling was further confirmed in the patients with intestinal bile acid deficiency (IBAD).

## 2 MATERIALS AND METHODS

### 2.1 Patients and samples

Between 2016 and 2018, 24 jejunal specimens from IBAD patients and 21 normal controls were obtained from patients who underwent pancreaticoduodenectomy at the First Affiliated Hospital of Zhejiang University, School of Medicine (Hangzhou, China). The diagnosis of IBAD depended on the level of blood total bilirubin and the presence of biliary tract obstruction. The detailed clinical information was summarized in Table S1. Informed consent was obtained from all patients, and this project was approved by the Ethics Committee of the First Affiliated Hospital of Zhejiang University, School of Medicine. Clinical data on another 306 patients with or without IBAD were collected for further analysis.

### 2.2 Cell culture, transfection and biochemical regents

The human intestinal epithelial cells (HIECs) and rat intestinal epithelial cell line 6 (IEC‐6) were incubated in RPMI 1640 and DMEM medium, respectively, supplemented with 10% foetal bovine serum and maintained in standard culture conditions. The detailed methods of cell culture and transfection, and the information of biochemical regents are shown in Data S1.

### 2.3 2‐NBDG uptake experiments

2‐NBDG, a fluorescent tracer, was used for monitoring glucose uptake into living cells. HIECs were evenly seeded into plates, grown to 80% confluence and incubated with 2‐NBDG (100 μmol/L) in the presence or absence of other reagents at 37°C for the indicated times. Then, the mean fluorescence intensity (MFI) was quantified by flow cytometry (Canto II, BD) or a multifunctional microplate reader (BMG Labtech,) with the excitation wavelength set at 488 nm and emission wavelength set at 540 nm.

### 2.4 Metabolic flux measurements

The cellular glucose metabolism was evaluated with a Seahorse XF96 Extracellular Flux Analyzer (Agilent Technologies) following the manufacturer's instructions. Briefly, HIECs were plated in Seahorse XF96 cell culture microplates at 1 × 10^4^ cells per well and cultured for 24 hours. Then, the medium was replaced with Seahorse XF DMEM medium containing 25 mmol/L glucose, 2 mmol/L L‐glutamine and 1 mmol/L sodium pyruvate and incubated in CO_2_‐free incubator at 37℃ for 45 minutes. Oxygen consumption rate (OCR) and extracellular acidification rate (ECAR) were measured using the Seahorse system.

### 2.5 Animals

Male C57BL/6 mice aged 8 weeks were purchased from Shanghai Experimental Animal Center (Shanghai, China) and housed in a specific pathogen‐free facility with adequate food and water. All animal experiments were performed in accordance with the principles of Chinese Council on Animal Care.

### 2.6 IBAD (Bile duct ligation, BDL) mice model

The operative procedures for BDL were performed as described previously.^24^, ^25^

### 2.7 Ussing chamber experiments

The detailed experimental procedures were as previously described. ^24^, ^25^ Briefly, mice were fasted for 12 hours and killed. The proximal jejunum was dissected and mounted in the Ussing chambers. DMSO (vehicle) or chenodeoxycholic acid (CDCA) was added to the mucosal Krebs‐Ringer solution 15 minutes before challenge with 20 mmol/L D‐glucose. The electrogenic glucose transport (ΔIsc) was monitored for further analysis.

### 2.8 BBM vesicle preparation

Fasted mice were anaesthetized and subjected to laparotomy. Three jejunal loops (3 cm long) were ligated and perfused with 0.5 mL of Krebs‐Ringer solution without (control) or with CDCA. After in situ incubation for 10 minutes, 0.5 mL D‐glucose solution was injected into the lumen for another 10 minutes, and the loops were excised and opened along the mesenteric border. The BBM vesicles were scraped off with a glass blade for the Western blot analysis.

### 2.9 Intestinal permeability and 2‐NBDG transport

The procedures of intestinal permeability and 2‐NBDG transport assays are described in detail in Data S1.

### 2.10 Glucose tolerance test

The blood glucose measurements were performed with a standard Accu‐Check glucometer (Roche Diagnostics, Manheim, Germany). After a 12 hours overnight fast, the mice were treated with CDCA via gavage for 10 minutes, followed by an oral administration of D‐glucose. The blood glucose levels were determined from tail vein samples before (0 minute) and 15, 30, 45 and 60 minutes after D‐glucose administration.

### 2.11 Co‐Immunoprecipitation (Co‐IP) and Immunofluorescence (IF)

The methods of Co‐IP and IF are detailed in Data S1.

### 2.12 Other standard methods

RNA isolation, quantitative reverse transcription PCR (qRT‐PCR), Western blot (WB) and immunohistochemistry (IHC) were performed with the standard method and previously described. ^24^, ^25^ For membrane, nuclear and cytoplasmic extraction, cells were subjected to Mem‐PER and NE‐PER Extraction Reagents Kit (89 842 and 78 833, Thermo) according to the manufacturer's instruction. The PCR primers and antibodies are shown in Tables S2 and S3.

### 2.13 Statistical analysis

Statistical analyses were performed with GraphPad Prism V5.01 software. All results were expressed as the means ± SD (standard deviation) using Student's t test between two groups and one‐way analysis of variance (ANOVA) when comparing more than two groups. Each experiment was independently repeated at least three times. *P* < .05 was considered as statistically significant.

## 3 RESULTS

### 3.1 Effects of BAs on glucose homeostasis in the intestine

**Figure 1:**
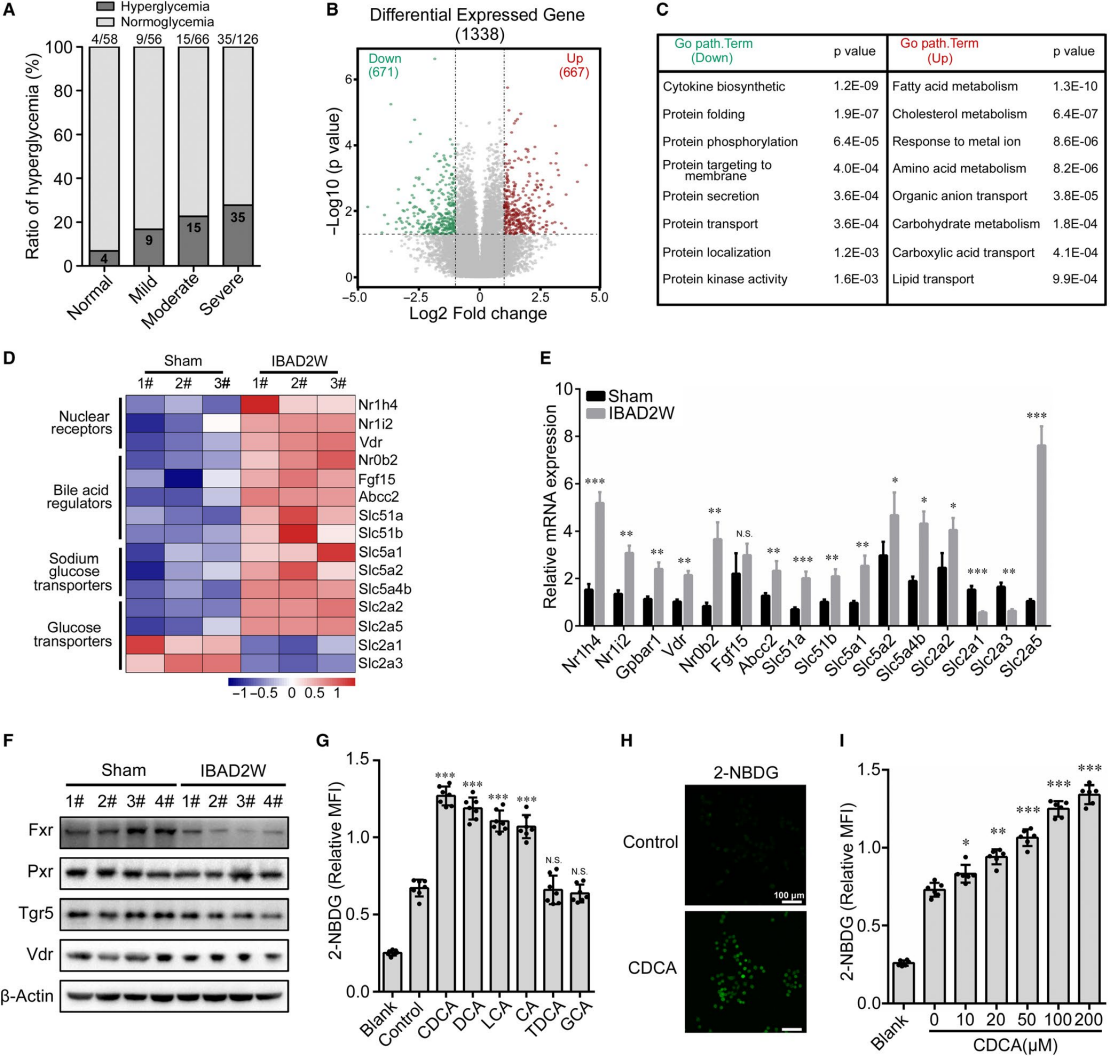
Effects of BAs on glucose homeostasis in the intestine. A, The ratios of aberrantly elevated serum glucose levels in normal and IBAD patients (Total serum bilirubin: mild deficiency, 34.2 ~ 85.5; moderate deficiency, 85.5 ~ 171; severe deficiency, >171 µmol/L). B‐D, Microarray analysis and metabolic studies of jejunum segments from 14‐day IBAD mice compared with the sham (control) group (n = 3 for each group). Volcano plots (B), Gene Ontology analysis of differentially expressed genes (C) and Heat maps (D). E, F, The analysis of qRT‐PCR (E) and Immunoblot (F) in jejunal tissues from 14‐day IBAD mice and sham mice. G, 2‐NBDG uptake in HIECs treated with different bile acids (each 100 μmol/L) for 30 min. H, Effect of CDCA (100 μmol/L) on 2‐NBDG uptake in HIECs. I, 2‐NBDG fluorescence intensity was quantified using a multimode reader. HIEC was treated with different concentrations of CDCA for 30 min in the presence of 2‐NBDG. Date are expressed as mean ± SD;^＊^ *P* ˂ .05, ^＊＊^ *P* ˂ .01, ^＊＊＊^ *P* ˂ .001, NS not statistically significant

The patients with IBAD secondary to biliary obstruction such as cholelithiasis or tumours from pancreatic head, the end of biliary duct and duodenal papilla, are often accompanied by severe disturbance in the lipid and glucose metabolism. Clinically, we found that the ratios of aberrantly elevated serum glucose levels were substantially increased as the disease progressed and nearly reached 30% in patients with severe IBAD (Figure 1A). To explore the global effects of BAs in the intestine, the mRNA levels of jejunum from the mice 14 days after IBAD and sham (control) were compared by microarray analysis. The expression of 667 and 671 genes was significantly up‐regulated and down‐regulated, respectively (Figure 1B). Gene Ontology analysis showed that differential genes with high significance were involved in carbohydrate, fatty acid and cholesterol metabolism, as well as anion transport (Figure 1C). Intestine bile deficiency resulted in a substantial up‐regulation of genes related to BA receptors, BA regulators and glucose transporters (Figure 1D). Confirming the microarray data, the expression of intestinal genes encoding BA receptors, that is Nr1h4, Nr1i2, Vdr and Gpbar1, and their regulated genes, that is Nr0b2, Abcc2, Slc51a and Slc51b, were up‐regulated in the IBAD mice. Glucose transporter genes, including Slc5a1, Slc5a2, Slc5a4b, Slc2a2 and Slc2a5, were also up‐regulated, except for Slc2a1 and Slc2a3 (Figure 1E). However, markedly reduced FXR, slightly decreased TGR5 (Gpbar1) and vitamin D receptor (VDR, Vdr), and not changed pregnane X receptor (PXR, Nr1i2) protein levels were observed in IBAD mice (Figure 1F). Subsequently, we found that the levels of protein ubiquitination were significantly up‐regulated in the jejunal tissues of IBAD mice (Figure S1A), indicating that BAs affect the protein expression of FXR through post‐transcriptional modifications.

To determine whether BAs affect glucose uptake in intestinal epithelial cells, we carried out a series of 2‐NBDG uptake measurements in vitro. The results showed that the 2‐NBDG uptake in the HIECs occurred within 5 minutes, increased and reached a plateau phase at 30 minutes (Figure S1B). Meanwhile, various BAs, including CDCA, deoxycholic acid (DCA), lithocholic acid (LCA), Cholic acid (CA), rather than taurodeoxycholic acid (TDCA) and glycocholic acid (GCA), enhanced the capacity of 2‐NBDG uptake to varying extents (Figure 1G). Indeed, CDCA, DCA, LCA and CA could lead to the activation of FXR. Next, CDCA was selected for the further experiments because it is a potent natural activator of FXR.^26^ The results showed that CDCA facilitated 2‐NBDG uptake in HIECs (Figure 1H), increased by almost threefold more than 2‐NBDG alone (Figure S3C,D) and occurred in a dose‐dependent manner (Figure 1I). However, 2‐NBDG uptake in HIECs remained almost unchanged when pre‐treated with CDCA for different concentrations or times (Figure S1E‐H), indicating that glucose uptake in intestinal epithelial cells is a transient process and is independent of the pre‐treatment time of BAs.

### 3.2 BA‐mediated glucose uptake depends on the FXR and GLUT2

**Figure 2:**
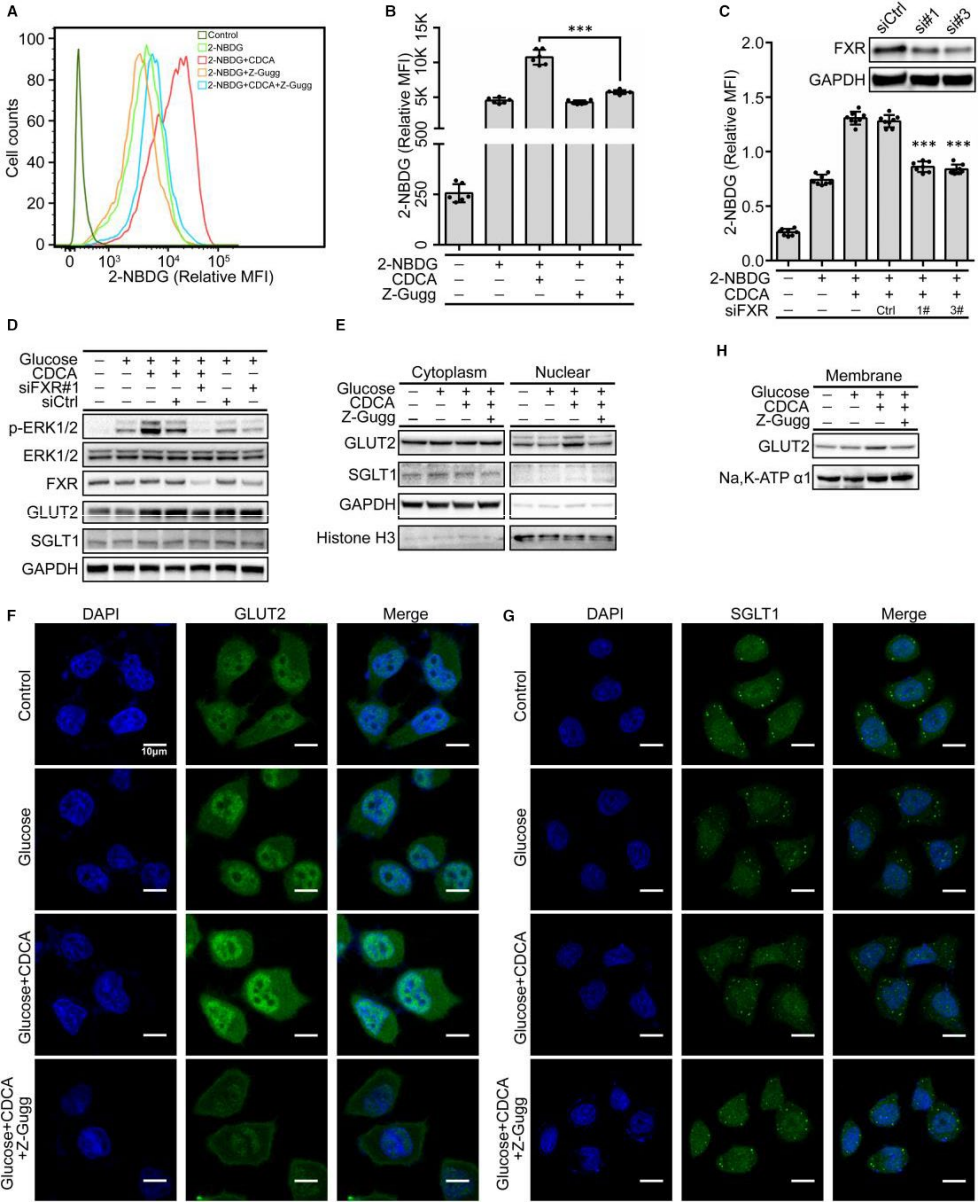
Bile acid facilitates glucose uptake by FXR‐mediated GLUT2. A, B, 2‐NBDG fluorescence intensity was analysed by flow cytometry (A) and quantitation of flow cytometry data (B). HIECs were pre‐treated with Z‐Gugg (10 µmol/L) for 1 h before the challenge with CDCA (100 μmol/L) and 2‐NBDG for 30 min. C, Analysis of 2‐NBDG uptake using a multimode reader in FXR‐silenced HIECs treated with CDCA (100 μmol/L) for 30 min. D, Immunoblot analysis of the indicated proteins in FXR‐silenced HIECs treated with glucose (25 mmol/L) for 30 min in the presence or absence of CDCA (100 μmol/L). E‐G, Analysis of GLUT2 and SGLT1 by Western blot in cytoplasmic and nuclear lysates (E), GLUT2 (F) and SGLT1 (G) by IF staining in the HIECs treated with glucose (25 mmol/L), CDCA (100 μmol/L) and Z‐Gugg (10 μmol/L) for 1 h. H, Immunoblot analysis of membrane GLUT2 in the HIECs treated with glucose (25 mmol/L), CDCA (100 μmol/L) and Z‐Gugg (10 μmol/L) for 1 h. All values are presented as the mean ± SD. ^＊^ *P* ˂ .05, ^＊＊＊^ *P* ˂ .001

Based on the above observations and the function of FXR in glucose metabolism, we suggested that FXR is involved in BA‐mediated glucose uptake. Intriguingly, pre‐treatment with CDCA for different times in HIECs did not induce any changes in the protein levels of FXR, GLUT2 and SGLT1 (Figure S2A). However, blocking FXR activity using Z‐Guggulsterone (Z‐Gugg), an FXR‐specific antagonist, significantly decreased 2‐NBDG uptake induced by CDCA (Figure 2A,B). Similar results were obtained in FXR‐silenced HIECs (Figure 2C), suggesting that FXR plays an essential role in BA‐mediated glucose uptake.

To determine how the FXR affects glucose uptake in HIECs, whole cell lysates were analysed to detect FXR, GLUT2 and SGLT1 by Western blotting. Interestingly, Z‐Gugg did not alter the expression of GLUT2 and SGLT1 in the absence of glucose (Figure S2B). However, in the presence of glucose, the GLUT2 was increased in HIECs when treated with CDCA for 1 hour (Figure 2D). Meanwhile, the combination of CDCA and glucose significantly increased the GLUT2 rather than SGLT1 in the nucleus, which could be blocked by Z‐Gugg (Figure 2E). This finding was also observed by IF, which displayed that CDCA induced an increase of GLUT2 in the nucleus in the presence of glucose, while SGLT1 remained localized on the plasma membrane (Figure 2F,G). Meanwhile, the elevated GLUT2 was also observed on the cell membrane in CDCA plus glucose group (Figure 2H). Hence, we speculate that BA‐mediated FXR may induce the transport of increased GLUT2 from nucleus to cell membrane for facilitating glucose uptake.

### 3.3 FXR‐mediated glucose uptake involves ERK1/2 phosphorylation

**Figure 3:**
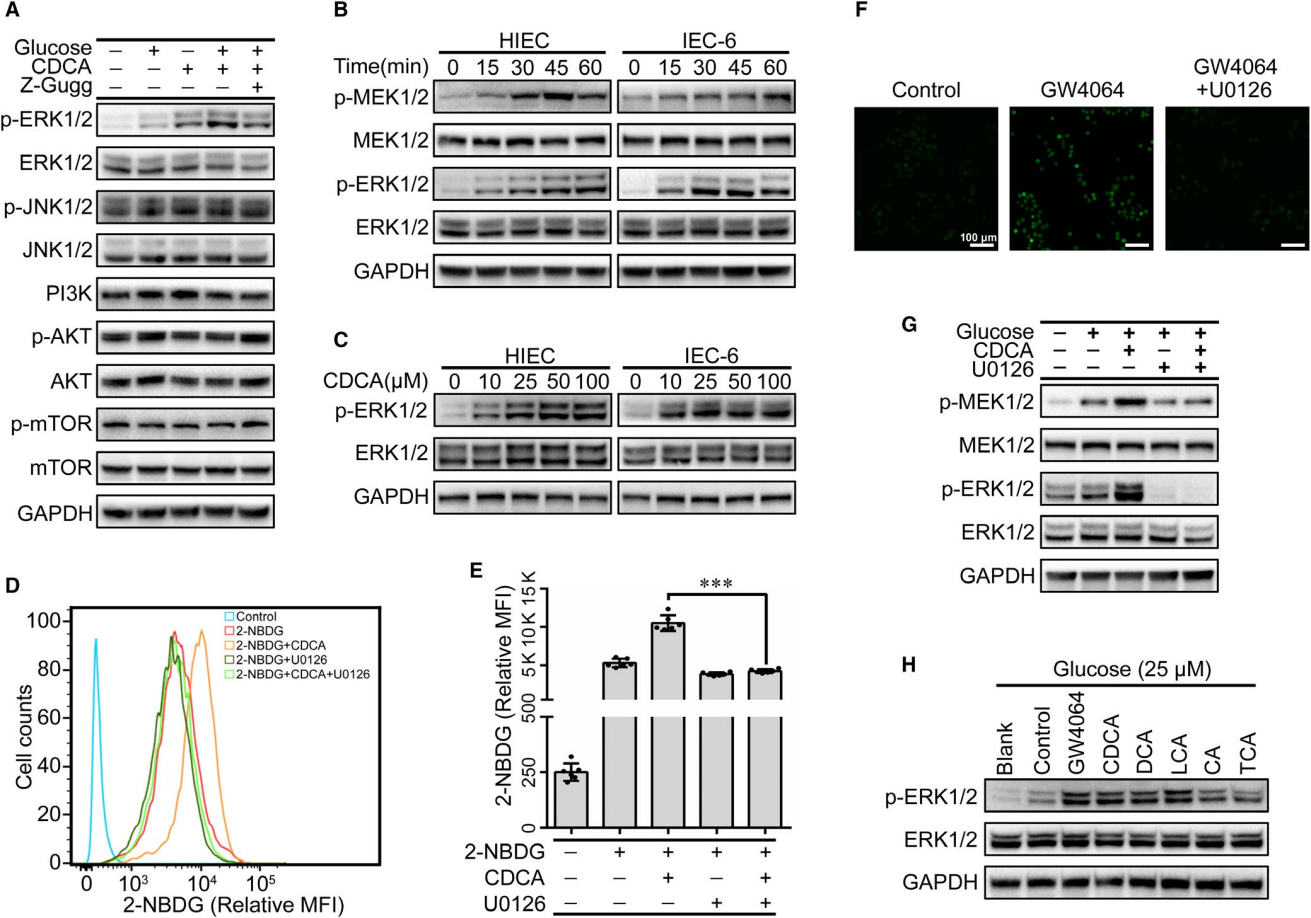
FXR‐mediated glucose uptake involves ERK1/2 phosphorylation. A, Western blot analysis of indicated protein levels in HIECs treated with glucose (25 mmol/L), CDCA (100 μmol/L) or Z‐Gugg (10 μmol/L) for 30 min. B, Activated MEK/ERK pathway was verified by Western blot analysis in HIECs and IEC‐6 cells treated with glucose (25 mmol/L) for different time. C, Immunoblot analysis of ERK1/2 and p‐ERK1/2 in HIECs and IEC‐6 cells treated with different concentrations of CDCA for 30 min. D, E, The effect of U0126 on 2‐NBDG uptake was analysed by flow cytometry (D) and quantitation of flow cytometry data (E). HIECs were treated with CDCA (100 μmol/L), U0126 (10 µmol/L) and 2‐NBDG for 30 min. F, Effect of GW4064 (10 μmol/L) on 2‐NBDG uptake in HIECs. G, Western blot analysis for p‐ERK1/2 and p‐MER1/2 in HIECs pre‐treated with U0126 (10 μmol/L) for 1 h, followed by 25 mmol/L glucose with or without CDCA (100 μmol/L) for 30 min. H, Immunoblot analysis of ERK1/2 and p‐ERK1/2 in HIECs treated with GW4064 (10 μmol/L) and individual BAs (100 μmol/L) in the presence of glucose (25 mmol/L). All values are presented as the mean ± SD. ^＊＊＊^ *P* ˂ .001

BAs play an important role in the regulation of multiple protein kinase signalling pathways,^27^ so we speculated whether FXR activates intracellular signalling pathways such as JNK1/2, ERK1/2 or AKT, which in turn enhanced glucose uptake in the HIECs. Remarkably, glucose only resulted in the phosphorylation of ERK1/2, which could be further increased by CDCA and partially blocked by Z‐Gugg (Figure 3A), indicating that ERK1/2 is a critical downstream target of FXR. This effect was further supported in IEC‐6 cells (Figure S2C). Furthermore, we found that glucose could induce ERK1/2 phosphorylation in a time‐dependent manner (Figure 3B), and CDCA‐induced activation of ERK1/2 occurred in a dose‐dependent manner and at μmol/L levels (Figure 3C). To further confirm the effect of ERK signalling on glucose uptake in HIECs, U0126, a MEK‐specific inhibitor, was used to block the activation of ERK1/2. As shown in Figure 3D,E, the 2‐NBDG uptake was reduced when pre‐treated with U0126 prior to CDCA for 1 hour. U0126 also decreased the 2‐NBDG uptake induced by GW4064, an FXR‐specific agonist (Figure 3F). Further Western blot analysis showed that U0126 completely blocked CDCA‐induced ERK1/2 phosphorylation (Figure 3G). Meanwhile, in addition to CDCA, other FXR activators, including GW4064, DCA, LCA, CA and TCA, also enhanced glucose‐induced ERK1/2 phosphorylation in the HIECs (Figure 3H), suggesting that the MEK/ERK signalling pathway is essential for the FXR‐mediated glucose uptake.

### 3.4 FXR regulates ERK1/2 phosphorylation through its interaction with S1PR2

**Figure 4:**
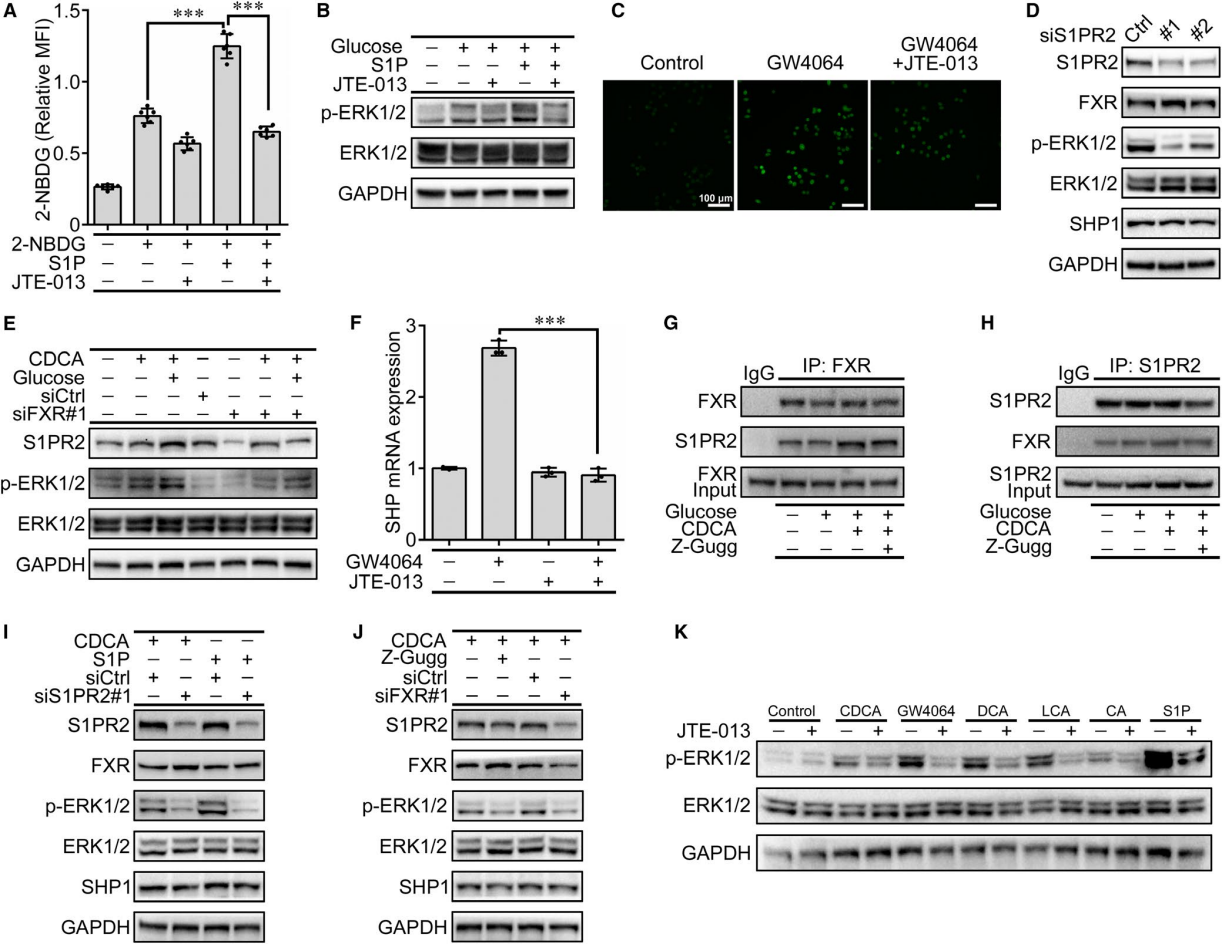
FXR regulates ERK1/2 activation through S1PR2. A, B, The effects of S1PR2 on 2‐NBDG uptake (A) and p‐ERK1/2 (B) in HIECs treated with S1P (100 nmol/L) or JTE‐013 (10 µmol/L) for 30 min. C, 2‐NBDG uptake in HIECs treated with GW4064 (10 μmol/L) and JTE‐013 (10 µmol/L) for 30 min. D, Immunoblot analysis of the indicated proteins in S1PR2‐silenced HIECs. E, Effects of FXR on S1PR2, ERK1/2 and p‐ERK1/2 were determined by Western blot in the FXR‐silenced HIECs treated with CDCA (100 μmol/L) or glucose (25 mmol/L) for 30 min. F, SHP mRNA levels induced by GW4064 (10 μmol/L) were determined by qRT‐PCR in HIECs treated with or without JTE‐013 (10 μmol/L) for 1 h. G, H, The interaction of FXR and S1PR2 was determined by Co‐IP in the HIECs treated with glucose (25 mmol/L), CDCA (100 μmol/L) and Z‐Gugg (10 μmol/L) for 1 h. I, J, Immunoblot analysis of the indicated proteins in S1PR2‐ or FXR‐silenced HIECs. The cells were treated with CDCA (100 μmol/L) for 30 min after challenge with 100 nmol/L S1P (I) or 10 μmol/L Z‐Gugg (J) for 1 h. K, Effect of JTE‐013 on ERK1/2 phosphorylation was determined by Western blot analysis. HIECs were treated with JTE‐013 (10 μmol/L) for 1 h, followed by individual BAs (CDCA, DCA, LCA, and CA; 100 μmol/L), GW4064 (10 μmol/L) or S1P (100 nmol/L) for 30 min. All values are presented as the mean ± SD.^＊＊＊^ *P* ˂ .001

Sphingosine‐1‐phosphate receptor 2 (S1PR2) is one of the five G protein‐coupled receptors for sphingosine‐1‐phosphate (S1P), a bioactive lipid molecule.^28^ BAs activate the ERK1/2 and AKT signalling pathways via S1PR2, which plays regulatory roles in glucose and lipid metabolism.^29^ Based on the above observations, we speculated that FXR may regulate ERK1/2 phosphorylation via S1PR2, which is involved in the glucose uptake. The results showed that S1P, a ligand of S1PR2, significantly induced 2‐NBDG uptake in HIECs, which was markedly inhibited by JTE‐013, a specific antagonist of S1PR2 (Figure 4A). Western blot analysis further identified that S1P‐activated ERK1/2 phosphorylation was blocked by JTE‐013 (Figure 4B). Meanwhile, JTE‐013 also inhibited 2‐NBDG uptake induced by FXR activation (Figure 4C), indicating that S1PR2 might be a downstream effector of FXR. Further studies showed that knockdown of S1PR2 using a specific siRNA failed to reduce the protein levels of FXR and SHP1 (Figure 4D). In contrast, the silencing of FXR in HIECs led to notable decreases in the protein levels of S1PR2 and p‐ERK1/2 (Figure 4E), indicating that S1PR2 acts as a downstream effector of FXR for regulating ERK1/2 phosphorylation. The activation of BAs receptor TGR5 can promote the internalization of S1PR2 in rat glomerulus mesangial cells.^30^ However, our results showed that the activation or inhibition of FXR failed to change the localization of S1PR2 in HIECs (Figure S3).

Small heterodimeric partner (SHP) is a direct FXR target gene that has been demonstrated to regulate BAs, glucose and lipid metabolism.^31^, ^32^ To further determine that S1PR2 is a downstream molecule of FXR signalling, we examined the effect of S1PR2 on SHP in the presence of GW4064. The results showed that GW4064 rapidly induced an increase in the SHP mRNA levels, which was significantly inhibited by JTE‐013 (Figure 4F). Subsequently, the interactions of FXR with S1PR2 were demonstrated by co‐immunoprecipitation, which showed that CDCA led to a profound increase in the interaction of FXR with S1PR2, while Z‐Guggulsterone decreased FXR binding to S1PR2 (Figure 4G‐H). Moreover, the CDCA/S1P‐mediated ERK1/2 was markedly inhibited when S1PR2 was silenced in the HIECs (Figure 4I). S1PR2 protein expression was also reduced when FXR was inhibited or silenced in the CDCA‐treated HIECs (Figure 4J). In addition, the phosphorylation of ERK1/2 induced by CDCA, GW4064, DCA, LCA, CA or S1P was blocked by JTE‐013 to different degrees (Figure 4K). These results support our hypothesis that S1PR2 acts as an intermediate regulator responsible for the FXR‐mediated ERK1/2 phosphorylation.

### 3.5 Effects of FXR on BA‐mediated glucose metabolism

**Figure 5:**
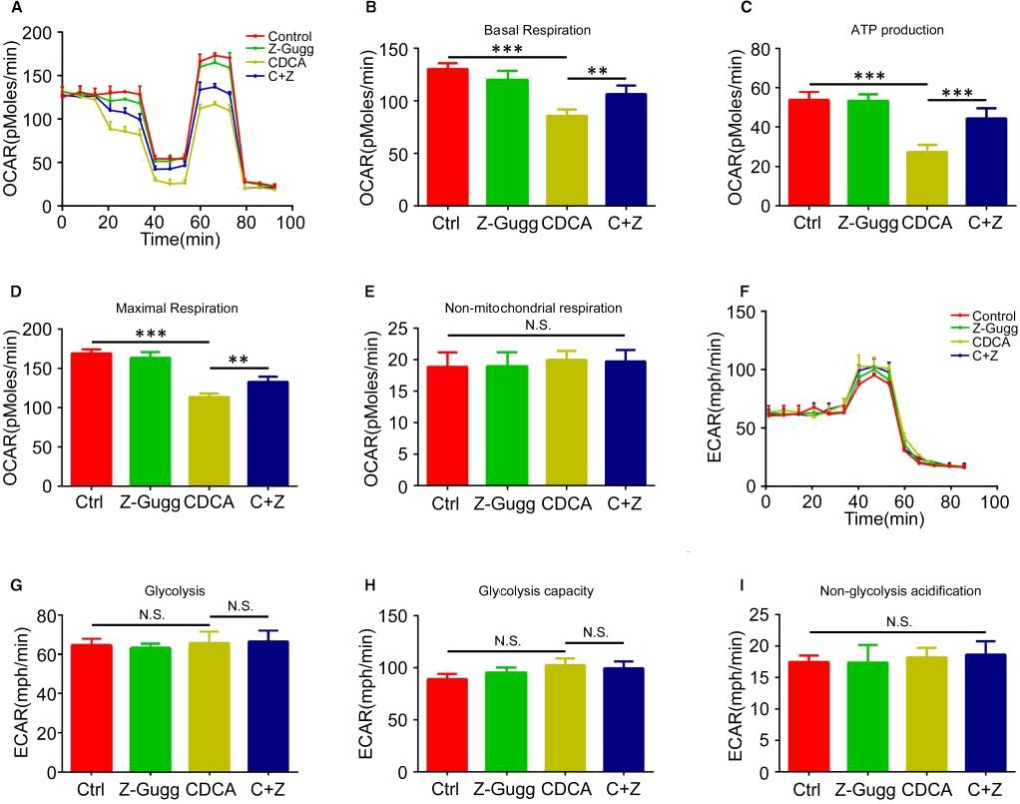
Effects of CDCA and FXR on glucose metabolism. The changes of glucose metabolism were elevated using a Seahorse system in HIECs treated with CDCA (100 μmol/L) and/or Z‐Gugg (10 μmol/L). A, OCR profile plot. B, Basal respiration. C, ATP‐linked respiration. D, Maximal respiration. E, Non‐mitochondrial respiration. F, ECAR profile plot. G, Glycolysis. H, Glycolytic capacity. I, Non‐glycolysis acidification. All values are presented as the mean ± SD. ^＊＊^ *P* ˂ .01, ^＊＊＊^ *P* ˂ .001, NS not statistically significant. C: CDCA; Z: Z‐Gugg

To further explore the effect of FXR on the BA‐mediated glucose metabolism, the metabolic stress response was examined using the Seahorse XF Cell Mito Stress Test kit and Glycolytic Rate Assay Kit. As shown in Figure 5A, CDCA significantly decreased the OCR that reflects the mitochondrial oxidative phosphorylation, and its action can be nullified when FXR was inhibited by the Z‐Gugg. Specifically, CDCA induced a decrease in the basal respiration, ATP production and maximal respiration, which were reversed in response to co‐treatment with Z‐Gugg (Figure 5B‐D). However, no significant difference in the non‐mitochondrial respiration was found between the groups (Figure 5E). Meanwhile, the Glycolysis Stress Test showed that CDCA and Z‐Gugg did not have any significant effects on the ECAR, an indicator of glycolytic flux, including glycolysis, glycolysis capacity and non‐glycolysis acidification (Figure 5F‐I). Overall, these results indicate that BA‐mediated FXR reduces the energy generation in HIECs via inhibition of oxidative phosphorylation.

### 3.6 FXR inhibits glucose transport by controlling GLUT2 abundance in the BBM

**Figure 6:**
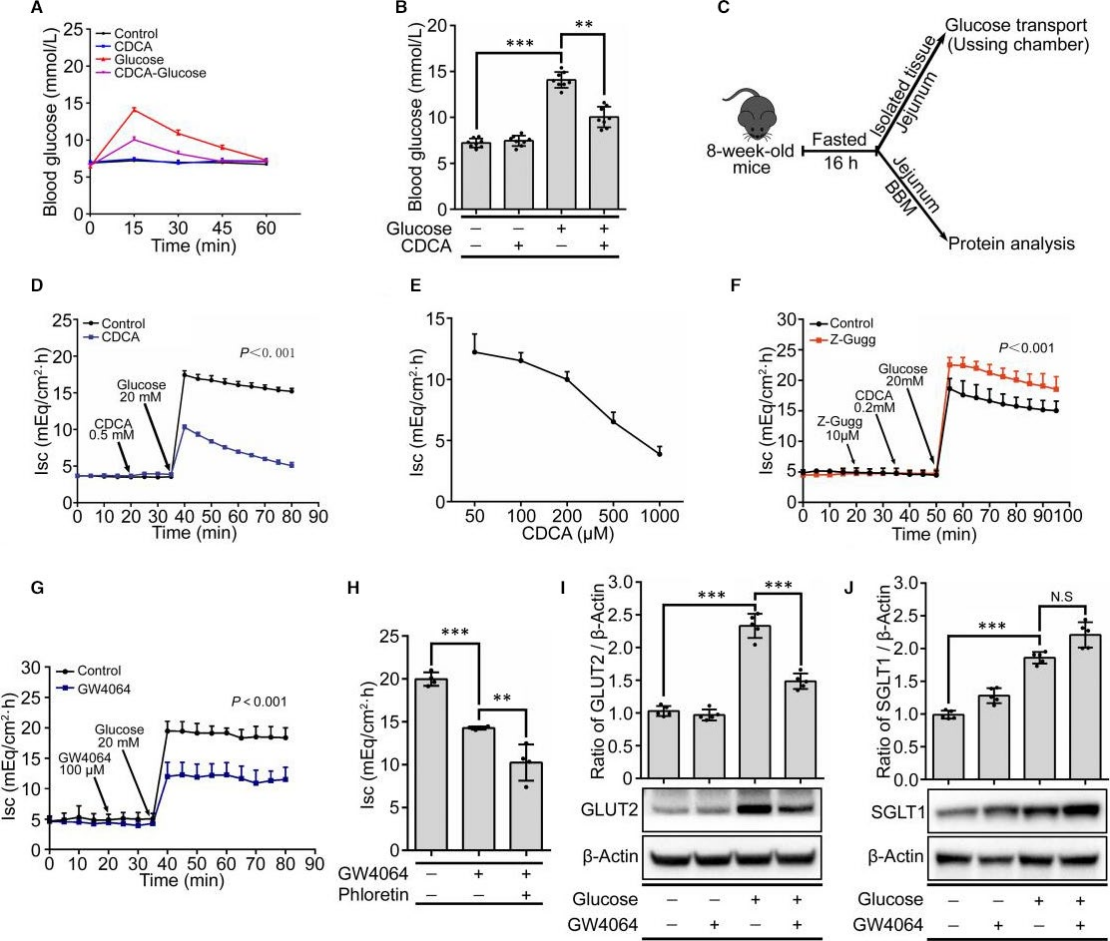
FXR inhibits intestinal glucose transport in vivo. A, Changes in blood glucose levels within 1 h after oral gavage of glucose (3 g/kg bodyweight) and/or CDCA(3 mg/kg bodyweight) in mice (n = 10 mice per group). B, Comparison of the net peak glucose increases between groups. C, Effects of CDCA on intestinal glucose transport were detected using the Ussing chamber system and analysed by the abundance of glucose transporters in the BBM by Western blotting. D, Effect of CDCA on glucose‐induced Isc (n = 6 mice per group). E, Glucose‐induced Isc after the addition of different concentrations of CDCA for 30 min (n = 5 or 6 per each group). F, Effect of Z‐Gugg on glucose‐induced Isc in the presence of CDCA (n = 6 mice per group). G, Effect of GW4064 on glucose‐induced Isc (n = 6 mice per group). H, The changes of glucose‐induced Isc in the presence of 10 μmol/L GW4064 and/or 1 mmol/L phloretin (n = 4 mice per group). I, J, Representative blots of GLUT2 (I) and SGLT1 (J) in jejunal BBM from mice treated with or without 20 mmol/L glucose and/or 500 μmol/L CDCA. Lower panels show representative immunoblots. Upper panels show the densitometric analysis of the blots (n = 5 mice per group). All values are presented as the mean ± SD. ^＊＊^ *P* ˂ .01, ^＊＊＊^ *P* ˂ .001, NS not statistically significant

To investigate the effects of BA in vivo, oral glucose tolerance tests were performed in fasted mice. The results showed that the blood glucose levels began to increase after the gavage administration of glucose and peaked within 15 minutes, whereas CDCA significantly inhibited the effect of glucose (Figure 6A, B). Subsequently, we evaluated the effects of BA and FXR on intestinal glucose transport using the Ussing chamber system and analysed the abundance of glucose transporters in the BBM (Figure 6C). As displayed in Figure 6D, the addition of glucose to the mucosal compartment rapidly induced an increase in the short circuit current (Isc, expressed as mEq.cm^‐2^.h^‐1^), reaching a plateau after 2 to 3 minutes. However, the glucose‐induced Isc was reduced following the addition of CDCA to the mucosal bath 15 minutes before the glucose. Meanwhile, the inhibition effects induced by CDCA occurred in a concentration‐dependent manner (Figure 6E). Consistently, CDCA inhibited 2‐NBDG transport from the mucosal to the serosal bath (Figure S4), suggesting that BA plays an inhibitory effects in the intestinal glucose transport. To test the role of FXR, the following FXR antagonist (Z‐Gugg) and agonist (GW4064) were used. The results showed that Z‐Gugg reversed the suppressive effect of CDCA on transepithelial transport of glucose (Figure 6F), while GW4064 significantly inhibited glucose‐induced Isc (Figure 6G). Thus, BA‐mediated FXR decreases the intestinal transepithelial transport of glucose from the lumen to the circulation, providing direct evidence of reduced blood sugar levels in mice following the oral gavage of CDCA.

Subsequently, we examined whether BA‐mediated FXR signalling reduces the transepithelial transport of glucose by modulating the intestinal permeability. Indeed, inulin test showed that GW4064 did not alter the permeability of the intestinal mucosa (Figure S5). To further understand the mechanism of the decreased glucose flux induced by FXR, we utilized phloretin, a GLUT2 transporter selective inhibitor. The results showed that the GW4064‐mediated transepithelial transport of glucose was further inhibited in the presence of phloretin (Figure 6H), suggesting that GLUT2 translocation is essential for the effect of FXR on glucose flux inhibition. Furthermore, the effect of FXR on GLUT2 was studied by Western blot analysis of the GLUT2 abundance in the BBM. The results showed that compared with the controls, glucose alone induced a 2.5‐fold increase in the GLUT2 levels, which could be reversed after ex vivo co‐incubation with GW4064 (Figure 6I). Because the functions of SGLT1 and GLUT2 in intestinal glucose transport closely coordinate and complement each other,^33^ the effect of FXR on SGLT1 was studied. As shown in Figure 6J, glucose also increased the abundance of SGLT1 in the BBM. However, the co‐incubation of glucose and GW4064 resulted in an almost onefold decrease in the abundance of GLUT2 in the BBM compared to glucose alone, whereas it only slightly increased the SGLT1 expression, indicating that GLUT2 plays a critical role in FXR‐mediated intestinal glucose transport.

### 3.7 **Intestinal FXR signal**l**ing is defective in IBAD patients**

**Figure 7:**
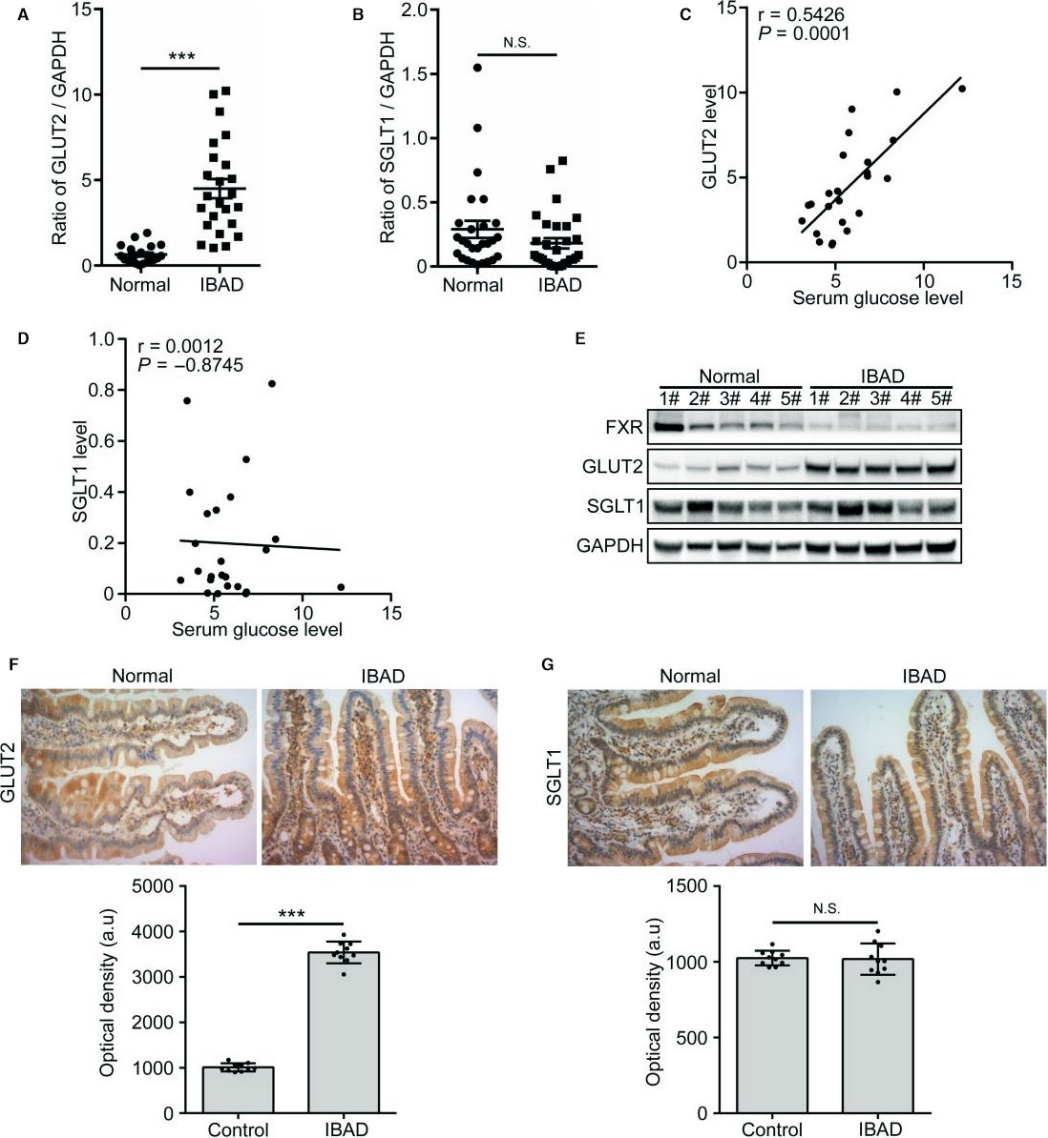
Intestinal FXR signalling is defective in IBAD patients. A, B, The mRNA levels of GLUT2 (A) and SGLT1 (B) in jejunal tissues from 24 IBAD patients and 21 normal patients (control). C, D, The correlation between the serum glucose levels and jejunal mRNA levels of GLUT2 (C) and SGLT1 (D) in 24 IBAD patients. E, Western blot analysis of indicated proteins in jejunal tissues from randomly selected IBAD patients and normal patients. F, G, GLUT2 (F) and SGLT1 (G) expression levels in jejunal mucosae from normal and IBAD patients. Upper panels are representative images (200×). Lower panels are the comparison of GLUT2 and SGLT1 expression levels. All values are expressed as mean ± SD. ^＊＊＊^ *P* ˂ .001, NS not statistically significant

Next, we examined the intestinal expression of GLUT2 and SGLT1 in 21 normal patients and 24 IBAD patients. The results showed that the mRNA levels of GLUT2 rather than SGLT1 were substantially increased in IBAD patients (Figure 7A, B). Additionally, the Pearson correlation analysis showed that the serum glucose levels were positively correlated with GLUT2 rather than SGLT1 mRNA levels in the IBAD patients (Figure 7C, D), indicating that GLUT2 may be a potential therapeutic target for the IBAD patients. The protein levels of FXR were markedly decreased, while GLUT2 was increased in the IBAD patients (Figure 7E), suggesting that FXR signalling is impaired in these patients. IHC further confirmed that GLUT2 was increased three‐ to fourfold and widely redistributed along the villi in the IBAD patients (Figure 7F), whereas no significant changes in the amount and distribution of SGLT1 were observed (Figure 7G).

## 4 DISCUSSION

Intestinal bile deficiency that occurs secondary to biliary obstruction is a common clinical presentation. Such deficiency initiates a series of pathophysiological events in the intestine, such as disorders of absorption and secretion, impaired mucosal barrier and dysbacteria, which are related to chronic diarrhoea, constipation, inflammatory bowel disease and even colorectal cancer.^34^, ^35^, ^36^ Our results showed that IBAD patients were more prone to develop hyperglycaemia, and the ratio of abnormally elevated blood glucose approximately reached 30% in severe IBAD patients, indicating that the amount of BAs in the intestine affects glucose metabolism in the body.

The microarray and metabolic analysis provided robust evidence that BAs play important regulatory roles in the intestinal glucose homeostasis. The alterations in BA receptors mRNA levels are not completely consistent with protein changes in the jejunum from IBAD mice. Indeed, nuclear receptors were not only regulated by post‐transcriptional modifications such as phosphorylation, acetylation and ubiquitination,^37^, ^38^, ^39^ but also affected under several conditions, including metabolic, hormonal and pathological situations.^12^, ^40^, ^41^ BAs affected protein expression by inhibiting ubiquitin proteasome degradation and inducing protein phosphorylation.^42^, ^43^ We demonstrated that IBAD can lead to significantly increased ubiquitination in jejunum tissues, suggesting that BAs regulate the expression of their receptors by post‐transcriptional modifications. Meanwhile, intestinal bile deficiency increased the expression of intestinal glucose transporters, suggesting that glucose transporters may play important roles in the BA‐mediated intestinal glucose homeostasis.

FXR has been previously implicated in lipid and glucose metabolism,^11^, ^12^ and FXR konckout mice showed a reduced and/or delayed intestinal glucose absorption.^44^ We next observed the effect of BAs on the glucose absorption in the intestinal epithelial cell. The results showed that only BAs, which can activate FXR, increased glucose absorption, indicating that FXR play a crucial role in the BA‐mediated intestinal glucose absorption. GLUT2 and SGLT1 are the main glucose transporters for intestinal glucose homeostasis between the intestinal epithelium and circulation. In vitro assays revealed that CDCA alone did not affect the total protein levels of FXR, GLUT2 or SGLT1. However, in the presence of glucose, CDCA‐mediated FXR markedly facilitated glucose uptake by increasing GLUT2 rather than SGLT1. Therefore, both FXR and GLUT2 are essential for BA‐mediated glucose uptake in the intestine.

FXR is known to be involved in several signalling pathways, including the ERK1/2, JNK1/2 and AKT pathways, to exert its biological effects.^45^, ^46^, ^47^ FXR activation stimulated intestinal secretion of FGF15/19, which can promote glucose uptake in adipocytes by activation of ERK1/2.^48^ Our findings regarding signalling cross‐talk also suggest that BA‐mediated FXR activates the MAPK pathway to affect intestinal glucose metabolism. The p‐ERK1/2 levels were increased by BA‐activated FXR signalling, which promoted glucose uptake to varying extents. Meanwhile, blocking the activation of ERK1/2 with U0126 resulted in a marked reduction in cellular glucose uptake, indicating that the FXR‐activated ERK1/2 may be central to BA‐mediated glucose uptake in vitro.

S1PR2 plays an important role in glucose, BA and lipid metabolism.^29^, ^49^ It is significantly up‐regulated in kidneys under diabetic conditions.^50^ We observed that S1P induced while JTE‐013 inhibited the glucose uptake in intestinal epithelial cells, suggesting that S1PR2 is involved in cellular glucose uptake. Conjugated bile acids can activate ERK1/2 and AKT through S1PR2 in rat hepatocytes.^29^ Our study suggested that the interaction of FXR with S1PR2 was enhanced by CDCA and decreased by FXR antagonist, indicating that FXR activity may affect the function of S1PR2. Meanwhile, knockdown of S1PR2 in HIECs decreased CDCA/S1P‐mediated ERK1/2 phosphorylation but not the FXR, whereas FXR silencing or inhibition of its activity decreased the expression of S1PR2 and p‐ERK1/2. Further, blocking S1PR2 signalling with JTE‐013 reduced not only the increase of SHP mRNA level induced by FXR activation, but also the ERK1/2 phosphorylation induced by S1P, GW4064 or many BAs that can activate FXR signalling. Altogether, these findings demonstrate that S1PR2, which is a key intermediator between FXR and ERK1/2, links FXR signalling to intestinal glucose homeostasis.

Once in the cell, the glucose is mainly used to provide metabolic energy. FXR has been shown to modulate hepatic carbohydrate metabolism during the fasting‐refeeding transition,^51^ and FXR activation also decreased glycolysis and ATP production in the enteroendocrine L‐cells.^52^ However, in intestinal epithelial cells, BA markedly reduced the ATP production via inhibition of oxidative phosphorylation rather than glycolysis, which can be rescued by FXR antagonist. Actually, intestinal glucose transport is a highly energy consuming process, which depends on the Na+/K+ATPase activity.^17^, ^53^ The reduced ATP production will directly affect the function of Na+/K+ATPase, resulting in the decrease of glucose transport. Thus, the cell will face the conflicting demands: there is a need to increase ATP by increasing glucose supply, but the very process of glucose transport utilizes ATP.^54^ Given that BAs and their receptors may participate in cellular glucose metabolism,^55^, ^56^ our future research will explore how the oxidative phosphorylation is affected by BA‐mediated FXR in the intestinal epithelial cells.

Glucose tolerance testing showed that BA markedly reduced the blood glucose levels in glucose‐loaded mice. Further studies revealed that BA‐activated FXR significantly inhibited the transepithelial transport of glucose in the intestine. Meanwhile, the inhibition is specific to glucose and is not associated with changes in intestinal permeability because inulin is not translocated from the luminal to serosal side in the activation of FXR. SGLT1 is primarily expressed in intestinal villi, and GLUT2 is considered to be located solely at the basolateral membrane.^57^ Indeed, FXR signalling triggers a molecular switch in the ratio of GLUT2 to SGLT1 in the BBM, thereby inhibiting intestinal glucose transport. Genetic studies have confirmed that variants of GLUT2 are involved in impaired fasting glucose and type 2 diabetes.^58^ We suggested that blocking GLUT2 with phloretin further inhibited the effects of FXR on glucose transport, indicating that GLUT2 is essential for FXR‐mediated intestinal glucose transport.

Clinically, patients with biliary obstruction often suffer from IBAD. The loss of FXR signalling impairs hepatic glucose homeostasis and decreases insulin sensitivity, while FXR activation improves hyperglycaemia and hyperlipidaemia in diabetic mice.^11^, ^12^ Consistently, impaired intestinal FXR signalling in IBAD patients leads to serious disturbances in the glucose homeostasis. In morbidly obese patients with insulin resistance, GLUT2 was mainly accumulated in apical and/or endosomal membranes of enterocytes.^59^ Our study demonstrated that increased GLUT2 abundance was observed in the BBM and positively correlated with the fasting plasma glucose levels, suggesting GLUT2 may represent a potential therapeutic target in IBAD patients with dysglycaemia.

In summary, this study clarifies the underlying mechanism of hyperglycaemia in IBAD patients and reveals a novel function of FXR in regulating the intestinal glucose homeostasis. Meanwhile, it uncovers a new therapeutic avenue for FXR agonist such as obeticholic acid in the treatment of IBAD‐related hyperglycaemia.

## CONFLICT OF INTEREST

The authors declare no conflicts of interest.

## AUTHOR CONTRIBUTIONS

PS, SZ and LZ conceived and designed the experiments; LZ, ZX and WS performed the experiments; LZ, ZX, SZ, ZL and GS helped with the human intestinal biopsy specimen; LZ, WS and XZ assisted with data analysis; LZ and PS wrote the manuscript. All authors have reviewed and approved the final manuscript.

## Supporting information

Supplementary Material

## Data Availability

All data included in this study are available upon reasonable request by contact with the corresponding authors.
