# Supplementary material for: A novel role for farnesoid X receptor in the bile acid‐mediated intestinal glucose homeostasis
Source: J Cell Mol Med. 2020 Oct 8;24(21):12848–61. doi: 10.1111/jcmm.15881 (PMC7686993; doi:10.1111/jcmm.15881)
Supplement: Supplementary file 1 — Supplementary Material [file JCMM-24-12848-s001.doc]

**A novel role for farnesoid X receptor in the bile acid-mediated intestinal glucose homeostasis**

Long Zhao^1,2,3,4,5^, Zefeng Xuan^1,2,3,4,5^, Wenfeng Song^1,2,3,4,5^, Shiyu Zhang^1,2,3,4,5^, Zequn Li^1,2,3,4,5^, Guangyuan Song^1,2,3,4,5^, Xingxin Zhu^1,2,3,4,5^, Haiyang Xie^1,2,3,4,5^, Shusen Zheng^1,2,3,4,5^, Penghong Song^1,2,3,4,5^

^1^Division of Hepatobiliary and Pancreatic Surgery, Department of Surgery, First Affiliated Hospital, School of Medicine, Zhejiang University, Hangzhou, China

^2^NHCPRC Key Laboratory of Combined Multi-organ Transplantation, Hangzhou, China

^3^Key Laboratory of the Diagnosis and Treatment of Organ Transplantation, CAMS, Hangzhou, China

^4^Key Laboratory of Organ Transplantation, Zhejiang Province, Hangzhou, China

^5^Collaborative Innovation Center for Diagnosis Treatment of Infectious Diseases, Hangzhou, China

**Correspondence:** Penghong Song and Shusen Zheng, Division of Hepatobiliary and Pancreatic Surgery, Department of Surgery, First Affiliated Hospital, School of Medicine, Zhejiang University, No.79 Qing chun Road, Hangzhou, 310003, China. E-mail: [songpenghong@zju.edu.cn](mailto:songpenghong@zju.edu.cn) (PS); shusenzheng@zju.edu.cn (SZ)

Zhao and Xuan contributed equally to this work.

**Supplemental Materials and Methods**

**Cell culture, transfection and biochemical regents**

The HIECs were gifted from the institute of Radiation Medicine, Fudan University (Shanghai, China), and the IEC-6 cells were obtained from American Type Culture Collection (Manassas, VA). HIECs were incubated in RPMI 1640 (Gibco, CA, USA) and IEC-6 were incubated in DMEM (Gibco, CA, USA), supplemented with 10% fetal bovine serum (Gibco, CA, USA), and maintained in standard culture conditions. All small interfering RNAs (siRNAs) were purchased from RiBoBio (Guangzhou, China). The siRNA and negative control siRNA were transfected into HIECs at 50 nM using Lipofectamine 3000 (Invitrogen, Carlsbad, CA), according to manufacturer’s protocol. Cells were analyzed and harvested 72 hrs after transfection. The siRNA sequences are as follows: NR1H4-#1, 5′-GTGGAACCATACTCGCAAT-3′; NR1H4-#3, 5′-TCCTCGTCATCCTATTATT-3′; S1PR2-#1, 5′-CATCCTCTGTTGCGCCATT-3′; S1PR2-#2, 5′-CCCGAAACAGCAAGTTCCA-3′. The bile acids (CDCA, DCA, LCA, CA, TCA, GCA, TDCA), Z-Guggulsterone (Z-Gugg, FXR antagonist) and Phloretin (GLUT2 inhibitor) were purchased from Sigma-Aldrich (St. Louis., MO, USA). 2-NBDG was purchased from Invitrogen (Carlsbad, CA, USA). S1P (S1PR2 ligand) was from Cayman Chemical (Ann Arbor, MI, USA). U0126 (MEK inhibitor), JTE-013 (S1PR2 antagonist) and GW4064 (FXR agonist) were obtained from Selleck Chemicals (Houston, TX, USA). FITC-inulin was purchased from TdB Consultancy (Uppsala, Sweden).

**Co-Immunoprecipitation (Co-IP)**

Co-IP was performed using a Dynabeads® Co-Immunoprecipitation Kit (14321D, Thermo) according to the manufacturer's instructions. Briefly, HIECs were harvested and lysed in ice-cold IP lysis buffer containing protease inhibitor cocktail (78430, Thermo) for 15 min. After centrifugation, the supernatant was incubated with the indicated antibody‐conjugated beads for 30 min. Then the beads were resuspended in Elution Buffer and incubated at room temperature for 5 min. Subsequently, the elute liquid was collected for further analysis by Western blotting.

**Immunofluorescence (IF) assays**

HIECs were fixed with 4% paraformaldehyde for 10 min, permeabilized with 0.1% Triton-X 100 for 10 min, blocked with 5% bovine serum albumin for 1 h at room temperature, and incubated overnight at 4 ℃ with primary antibodies targeting GLUT2 (Abcam, ab54460; 1:200) or SGLT1 (Sigma-Aldrich, SAB2700874; 1:200). Then, cells were washed three times with PBS, incubated with Alexa Flour 488- conjugated goat anti-rabbit secondary antibody (Invitrogen, Grand Island, NY) for 1 h at room temperature, rewashed with PBS and mounted with anti-fade mounting solution containing DAPI (Vector Labs, Burlingame, CA). The images were captured using OLYMPUS IX83-FV3000- OSR (Olympus Corporation, Japan).

**Intestinal permeability and 2-NBDG transport**

The effect of FXR on the intestinal permeability was determined by measuring the mucosal-to-serosal flux rate of the paracellular probe FITC-labeled inulin (MW = 4 kDa). In brief, mice were fasted for 12 hrs and killed. The jejunal sacs were dissected and mounted in the Ussing chambers. DMSO (vehicle) or GW4064 (10 µM) was added to the mucosal bath for 15 min, followed by the addition of FITC-inulin for 10 min. Then, the fluorescence intensity of serosal solutions was quantified by a multifunctional microplate reader (BMG Labtech, Ortenberg, Germany). Similar procedures were performed to detect the transepithelial transport of 2-NBDG.

**Supplemental Figure Legends**

**Supplemental Figure 1** Effects of BAs on ubiquitination and glucose uptake in the intestine. A, Western blot analysis of jejunal ubiquitination level in 14-day IBAD and Sham mice. B, 2-NBDG (100 μM) uptake in HIECs was analysed at different time points with a multimode reader. C, D, Analysis of 2-NBDG uptake in HIECs treated with 100 μM CDCA for 30 min by flow cytometry (C) and quantitation of flow cytometry data (D). E, 2-NBDG uptake was analysed using a multimode reader in HIECs pretreated with increasing concentration of CDCA for 24 hrs (0 μM is control). F-H, Analysis of 2-NBDG fluorescence intensity in HIECs pretreated with 100 μM CDCA for different time points by multimode reader (F), flow cytometry (G), and quantitation of flow cytometry data (H). Values are expressed as mean ± SD.^＊＊^*P* ˂ 0.01, ^＊＊＊^*P* ˂ 0.001, N.S. not statistically significant.

**Supplemental Figure 2** Effects of FXR on glucose transporters and ERK1/2 phosphorylation. A, Western blot was performed to detect the expression of FXR, GLUT2 and SGLT1 in HIECs treated with 100 μM CDCA for the indicated time. B, Western blot analysis of FXR, GLUT2 and SGLT1 in HIECs. Cells were treated with 100μM CDCA and 10 μM Z-Gugg for 30 min in the absence of glucose. C, The IEC-6 cells were treated with 25 mM glucose, 100μM CDCA and 10 μM Z-Gugg for 30 min, then the expression of ERK1/2 and p-ERK1/2 was analyzed by Western blot.

**Supplemental Figure 3** The effect of FXR on subcellular localization of S1PR2 in HIECs. The subcellular localization of S1PR2 was detected by IF in HIECs treated with 100 μM CDCA and 10 μM Z-Gugg for 30 min. Scale bar, 10 μm. DAPI: 4´,6-diamidino-2-phenylindole.

**Supplemental Figure 4** Effect of CDCA (500 μM) on transepithelial transport of 2-NBDG (100 μM) in the jejuna sacs from mice (n= 4 mice per group). Values are expressed as mean ± SD, ^＊＊＊^*P* ˂ 0.001.

**Supplemental Figure 5** Effect of GW4064 on Inulin permeability in jejunal sacs from mice (n= 4 mice per group). Values are expressed as mean ± SD, N.S. not statistically significant.

**Supplemental Figures**

**Supplemental Figure 1**

**
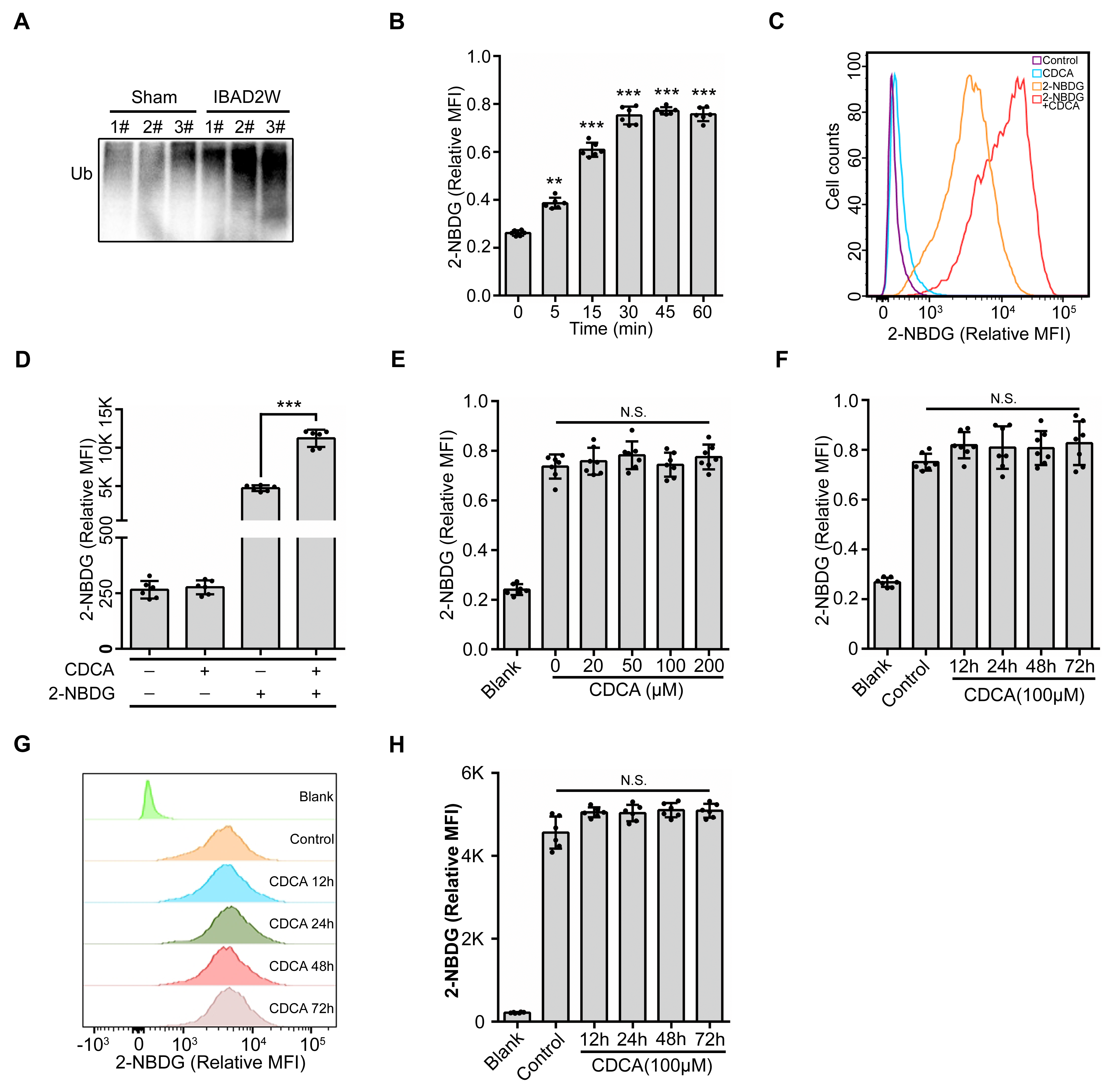
**

**Supplemental Figure 2**

**
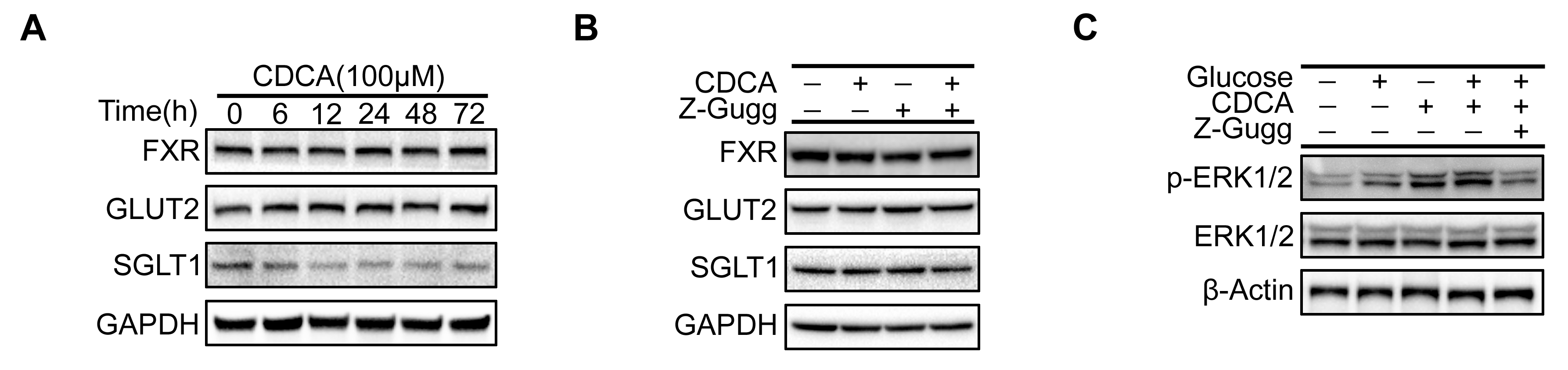
**

**Supplemental Figure 3**


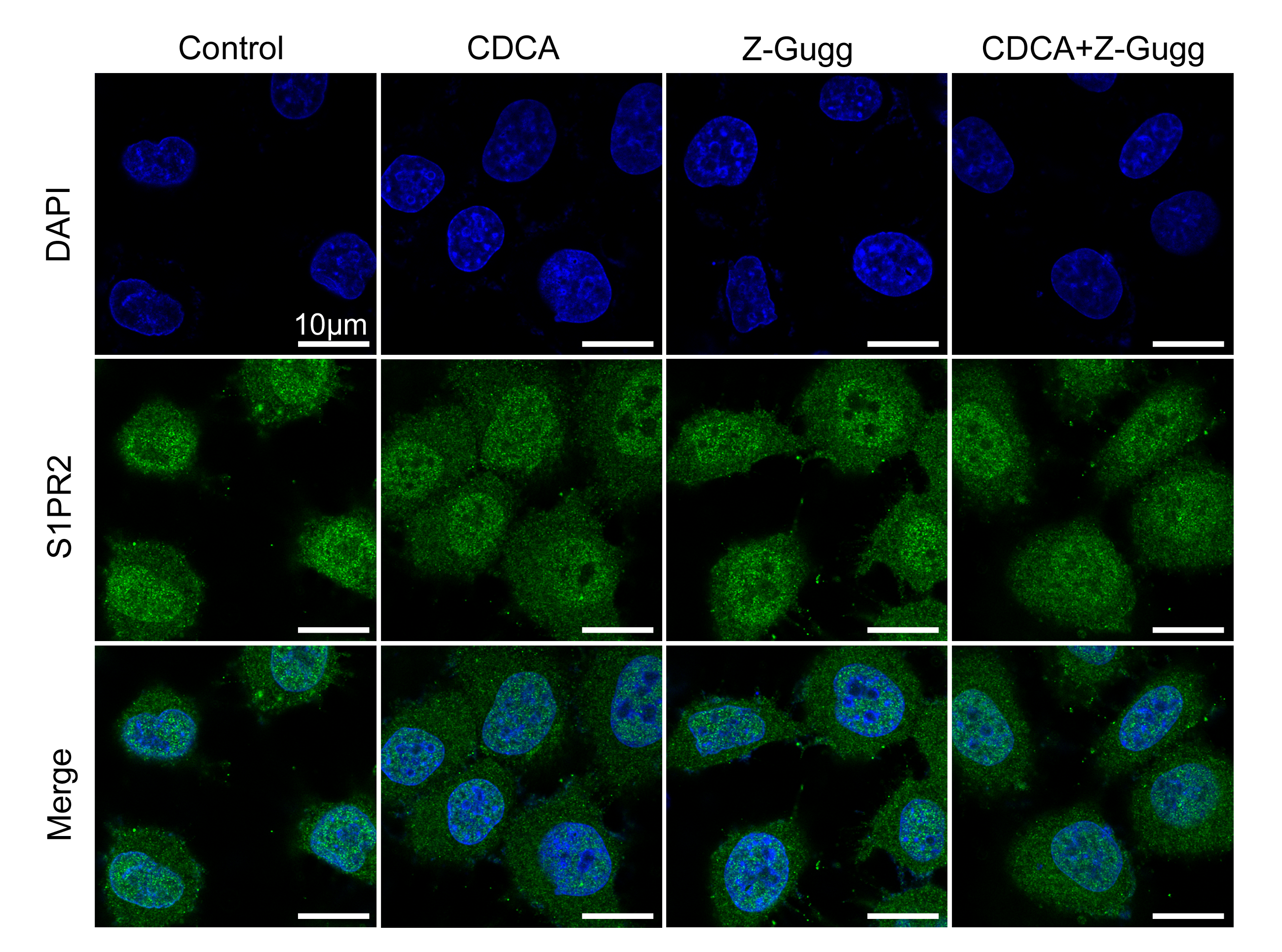


**Supplemental Figure 4**


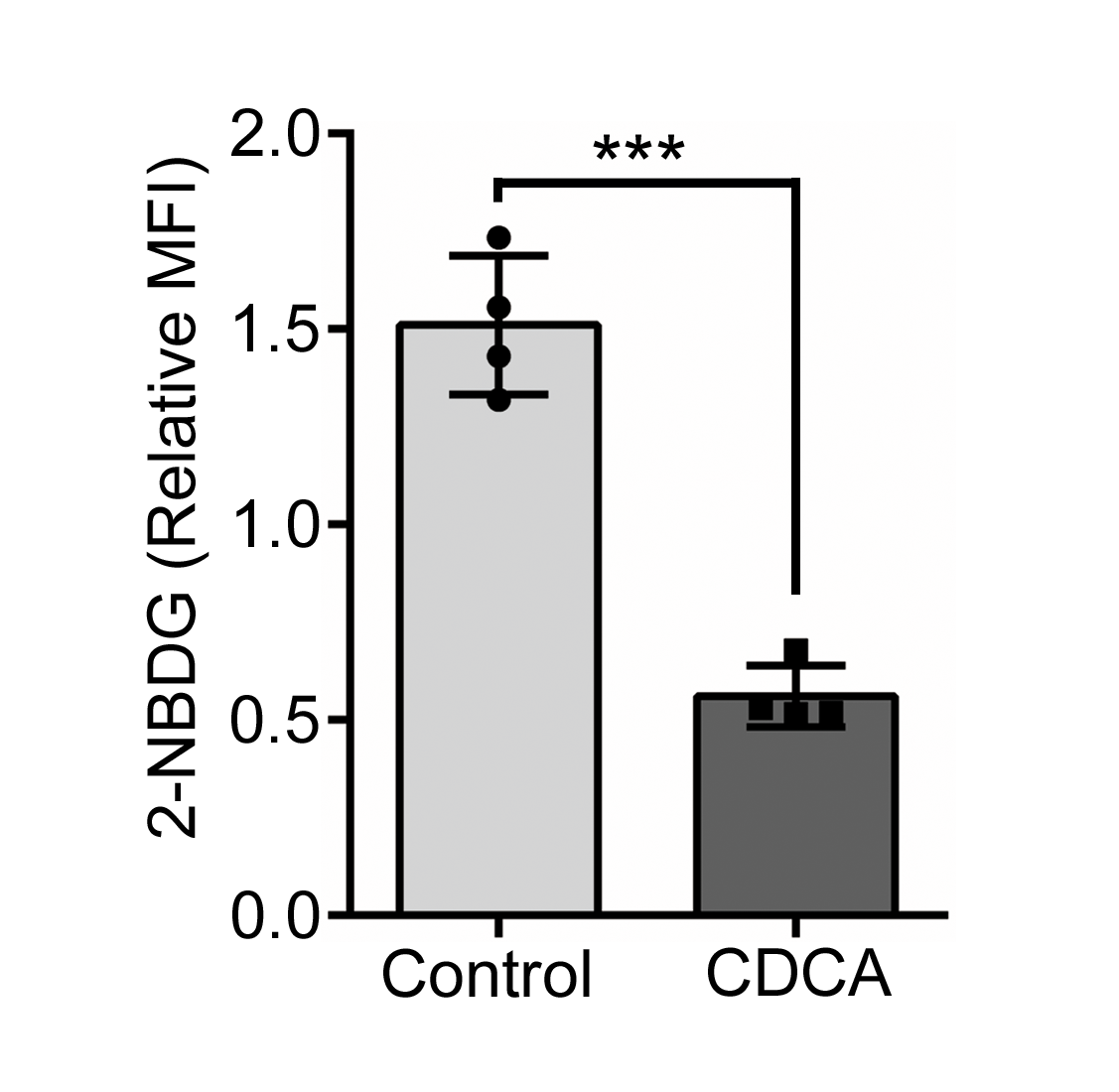


**Supplemental Figure 5**


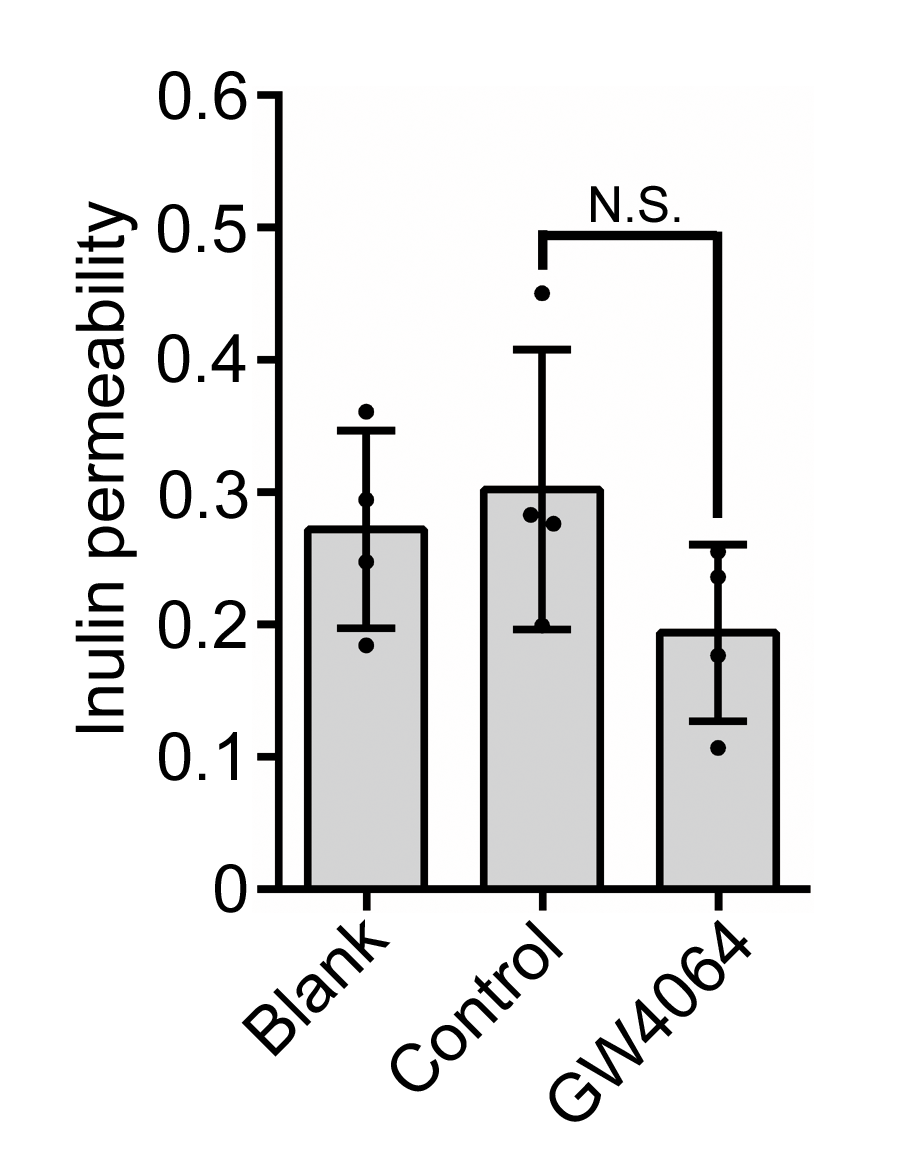


**Supplemental Table 1** Clinical characteristics in normal and IBAD patients who underwent pancreaticoduodenectomy

|  | Non-IBAD (21 patients) | | | | |  |  | IBAD(24 patients) | | | | |
| --- | --- | --- | --- | --- | --- | --- | --- | --- | --- | --- | --- | --- |
| Age | Sex | TB | DB | SGL | Diagnosis |  | Age | Sex | TB | DB | SGL | Diagnosis |
| 59 | M | 13 | 5 | 5.70 | PHD |  | 55 | M | 302 | 249 | 9.95 | DPT |
| 65 | F | 10 | 3 | 4.76 | PHC |  | 66 | M | 370 | 328 | 7.62 | DPT |
| 57 | M | 17 | 5 | 4.37 | PHD |  | 72 | F | 41.7 | 30.2 | 4.11 | EC |
| 59 | M | 11 | 4 | 8.90 | PHC |  | 68 | M | 512 | 355 | 12.18 | PHC |
| 73 | F | 18 | 5 | 4.46 | DPT |  | 78 | F | 162 | 118 | 6.84 | PHC |
| 36 | M | 10 | 4 | 5.31 | PHC |  | 71 | F | 335 | 253 | 7.81 | DPT |
| 60 | M | 18 | 8 | 2.99 | PHC |  | 63 | F | 190 | 157 | 5.22 | PHC |
| 47 | M | 17 | 4 | 4.73 | DPT |  | 64 | M | 474 | 346 | 7.40 | PHC |
| 67 | F | 13 | 7 | 5.35 | PHC |  | 53 | F | 81 | 55 | 5.94 | PHC |
| 70 | M | 15 | 7 | 4.78 | DPT |  | 59 | M | 201 | 174 | 3.12 | PHC |
| 83 | F | 18 | 9 | 5.30 | EC |  | 65 | F | 63 | 43 | 5.45 | PHC |
| 66 | M | 16 | 8 | 3.77 | EC |  | 81 | M | 469 | 357 | 8.85 | EC |
| 72 | F | 12 | 8 | 4.11 | DPT |  | 62 | F | 406 | 297 | 6.80 | EC |
| 69 | M | 11 | 6 | 4.80 | PHC |  | 47 | M | 183 | 137 | 5.77 | PHC |
| 46 | F | 16 | 9 | 4.78 | DPT |  | 62 | F | 166 | 134 | 5.66 | EC |
| 50 | F | 4.5 | 1.8 | 4.48 | PHC |  | 45 | M | 79 | 58 | 4.64 | EC |
| 67 | F | 8.3 | 3.4 | 5.06 | PHC |  | 67 | F | 319 | 227 | 7.95 | PHC |
| 45 | F | 13.2 | 3.8 | 5.06 | DPT |  | 70 | M | 477 | 309 | 7.82 | PHC |
| 43 | F | 9.3 | 3.5 | 5.10 | PHC |  | 64 | M | 89 | 70 | 8.28 | DPT |
| 49 | M | 15 | 5 | 5.15 | PHC |  | 65 | F | 69 | 45 | 6.35 | DPT |
| 50 | M | 10.1 | 3.8 | 4.44 | DPT |  | 66 | M | 123 | 80 | 3.48 | EC |
|  |  |  |  |  |  |  | 67 | F | 152 | 131 | 5.13 | EC |
|  |  |  |  |  |  |  | 64 | F | 184 | 129 | 8.48 | PHC |
|  |  |  |  |  |  |  | 64 | M | 122 | 81 | 3.63 | PHC |

IBAD, intestinal bile acid deficiency; DPT, duodenal papillary tumor; PHC, pancreatic head carcinoma; EC, extrahepatic cholangiocarcinoma; SGL, serum glucose level.

***Supplemental Table 2*** *Primer information*

| Gene | Forward (5’ to 3’) | Reverse (5’ to 3’) |
| --- | --- | --- |
| Human-GAPDH | GAGCCAAAAGGGTCATCATCT | TTCCACGATACCAAAGTTGTCA |
| Human-SLC2A2 | ACTCAACCAGCATTTTTCAGAC | CAAACAAACATCCCACTCATTC |
| Human-SLC5A1 | CTGGTTTTGGTGGTTGTGCT | GGGACAGAAGGGAAAGGTAGAC |
| Human-S1PR2 | CTGCTCAAGACGGTCACCAT | TAGTGGGCTTTGTAGAGGATCG |
| Human-NR0B2 | GCCCAGCATACTCAAGAAGATT | GACTCCAGACAGCATTGAAGC |
| mouse-β- actin | GCCTTCCTTCTTGGGTATGTT | GTCTTTACGGATGTCAACGGGTA |
| mouse-Nr1h4 | CTAATGAGGACGACAGCGAAG | CTGTTGGTCTGCCGTGAGTT |
| mouse-Nr1i2 | ACACAACTTTCTCCCACTTCAA | TGTGGCAGAAGAGGGATGAT |
| mouse-Vdr | TACACCCCCTCACTGGACAT | GATGACCTTTTGGATGCTGTAA |
| mouse-Gpbar1 | CCACACTGCTCTTCTTGCTG | CCAATGAGATGAGCGATAACAG |
| mouse-Nr0b2 | CTGGAGTCTTTCTGGAGCCTT | AGACTTCACACAGTGCCCAGT |
| mouse-Fgf15 | GAGGACCAAAACGAACGAAAT | GAGTAGCGAATCAGCCCGTAT |
| mouse-Abcc2 | CGTTTAGTTGGTATGACAGCAC | CTGGTGAGTGACTTGGCTTTA |
| mouse-Slc51a | GGACATAGCCCTCACCATCAT | AGCAGAACACAGATACCACCGT |
| mouse-Slc51b | GGAACTGCTGGAAGAAATGCT | TCTTTGTCTTGTGGCTGCTT |
| mouse-Slc5a1 | CTTTGAATGGAACGCCTTG | AAACCGCTTCCGCAGATAC |
| mouse-Slc5a2 | AACATTTACGCTTCGGTCATC | TCATTGCTCCCAGGTATTTGT |
| mouse-Slc5a4a | AGGAAGAAGGCATCAGAGAGAG | ACCAGTTGTCCACCTTGAGATA |
| mouse-Slc2a2 | TTGCTGGACGAAGTGTATC | GACTAATAAGAATGCCTGTGAC |
| mouse-Slc2a5 | CATCAAGAGGAGACAGGGGAG | GGTGTCATTGTAAAACTGCTGC |
| mouse-Slc2a1 | CGGGTATCAATGCTGTGTTCT | CTACAACAAACAGCGACACCAC |
| mouse-Slc2a3 | TCGCTGTTACTAAAGGATGACT | CCACAATAAACCAGGGAATG |

**Supplemental Table 3** Antibody information

| Antibody | Supplier | Antibody | Supplier |
| --- | --- | --- | --- |
| MEK1/2 | CST (8727) | AKT | Abcam (ab8805) |
| p-MEK1/2 | CST(9154) | p-AKT | Abcam (ab38449) |
| ERK1/2 | CST (4695) | FXR | Abcam (ab235094) |
| p-ERK1/2 | CST (8544) | FXR | Santa Cruz (sc-25309) |
| mTOR | CST (4517) | PXR | Abcam (ab192579) |
| p-mTOR | CST (5536) | TGR5 | Abcam(ab72608) |
| PI3K | CST (4249) | SHP1 | Abcam (ab32559) |
| VDR | CST (12550) | GAPDH | Abcam (ab8245) |
| Na,K-ATPaseα1 | CST (23565) | Histone H3 | Abcam (ab1791) |
| JNK1/2 | R&D (AF1387) | GLUT2 | Abcam (ab54460) |
| p-JNK1/2 | R&D (AF1387) | GLUT2 | Abcam (ab111117) |
| SGLT1 | Sigma(SAB2700874) | SGLT1 | Abcam (ab14686) |
| S1PR2 | Proteintech (21180-1-AP) |  |  |
